# Self-supervised learning for macromolecular structure classification based on cryo-electron tomograms

**DOI:** 10.3389/fphys.2022.957484

**Published:** 2022-08-30

**Authors:** Tarun Gupta, Xuehai He, Mostofa Rafid Uddin, Xiangrui Zeng, Andrew Zhou, Jing Zhang, Zachary Freyberg, Min Xu

**Affiliations:** ^1^ Department of Computer Science and Engineering, Indian Institute of Technology, Indore, India; ^2^ Department of Electrical and Computer Engineering, University of California, San Diego, San Diego, CA, United States; ^3^ Computational Biology Department, Carnegie Mellon University, Pittsburgh, PA, United States; ^4^ Irvington High School, Irvington, NY, United States; ^5^ Department of Computer Science, University of California, Irvine, Irvine, CA, United States; ^6^ Departments of Psychiatry and Cell Biology, University of Pittsburgh, Pittsburgh, PA, United States

**Keywords:** self-supervised learning, macromolecular structure classification, electron cryo tomograms, data augmentation, contrastive learning

## Abstract

Macromolecular structure classification from cryo-electron tomography (cryo-ET) data is important for understanding macro-molecular dynamics. It has a wide range of applications and is essential in enhancing our knowledge of the sub-cellular environment. However, a major limitation has been insufficient labelled cryo-ET data. In this work, we use Contrastive Self-supervised Learning (CSSL) to improve the previous approaches for macromolecular structure classification from cryo-ET data with limited labels. We first pretrain an encoder with unlabelled data using CSSL and then fine-tune the pretrained weights on the downstream classification task. To this end, we design a cryo-ET domain-specific data-augmentation pipeline. The benefit of augmenting cryo-ET datasets is most prominent when the original dataset is limited in size. Overall, extensive experiments performed on real and simulated cryo-ET data in the semi-supervised learning setting demonstrate the effectiveness of our approach in macromolecular labeling and classification.

## 1 Introduction

Cryo-electron tomography (cryo-ET) is a revolutionary imaging technology with notable applications in the field of cell and structural biology (Gan and Jensen, 2012; Lučić et al., 2013; Zhang, 2013). Our understanding of the structures and accompanying functions of key components of the cellular microenvironment have been significantly expanded by cryo-ET (Grünewald et al., 2003; Cyrklaff et al., 2005; Koning and Koster, 2009). Furthermore, cryo-ET has provided new insights into human disease states including mitochondrial diseases and, most recently, COVID-19 where the structure and function of SARS-CoV-2 was determined in infected host cells (Klein et al., 2020). Another major advantage of cryo-ET is that high-resolution 3D images of subcellular structures (e.g., organelles and macromolecules) are acquired in their near-native states in contrast to earlier approaches that require fixation, sectioning and dehydration steps that may distort or alter cellular architecture (Oikonomou and Jensen, 2017). The 3D images are referred to as tomograms and the small subvolumes of the tomograms that visualize individual macromolecule are termed subtomograms. 3D visualization by cryo-ET enables resolution of the structures of the subcellular components and their spatial interactions *in situ* within single cells.

To understand macromolecular interactions and dynamics, classifying individual macromolecular structures from the subtomograms is a crucial step (Murata and Wolf, 2018). The classification implies identifying the target macromolecules from subtomograms. However, due to the crowded and heterogeneous cellular environment, each subtomogram closely packs several neighboring potentially unrelated macromolecules alongside the target macromolecule of interest (Best et al., 2007). Thus, the closely packed structures in a single subtomogram makes macromolecular classification challenging (Best et al., 2007). Due to its resemblance to 3D image classification, several deep classification models have been deployed to perform macromolecular classification. VP-Detector (Hao et al., 2022), which uses 3D multiscale convolutional neural network, is one of the recent approaches for cryo-ET classification. However, most of these classification methods are supervised and sample-inefficient. For cryo-ET, availability of labelled data is limited due to the rigorous annotation process. Furthermore, the performance of deep supervised classification models relies on the number of labelled cryo-ET subtomograms (Frazier et al., 2017). One strategy to tackle the scarcity of labelled data is to generate simulated cryo-ET data on which supervised models will be trained - an approach used by several previous studies that simulated cryo-ET subtomogram data (Pei et al., 2016; Liu et al., 2020a,b). There is also a recent study using simulated data for supervised training followed by application to experimental data (Moebel and Kervrann, 2022). Nevertheless, models trained using simulated data often perform poorly when analyzing actual experimental data due to domain shift. In contrast, semi-supervised approaches have the capability to deal with lack of labelled data and avoid the problem of domain shift in simulated data. Thus several approaches (Yu et al., 2020; Du et al., 2021) have been developed that utilise both labelled and unlabelled data for subtomogram classification (Chapelle et al., 2009). However, the accuracy obtained from these approaches is yet to reach near the accuracy from supervised approaches. As a result, improvement of these semi-supervised approaches for subtomogram classification continues to remain a problem.

Recently, self-supervised learning (SSL) (Noroozi and Favaro, 2016; Pathak et al., 2016; Zhang et al., 2016; Komodakis and Gidaris, 2018) has been proven to be an effective unsupervised technique to learn data representations by solving auxiliary tasks on input data, which does not require any human-defined annotations. Contrastive Self-supervised Learning (CSSL) (Hadsell et al., 2006), as a subcategory of SSL, has been widely used to learn better representations of images and has been successful in achieving state-of-the-art results in various domains of image classification (He et al., 2020; Tian et al., 2019; Chen et al., 2020a; Caron et al., 2020; Misra and Maaten, 2020). CSSL learns image representations by optimizing the contrastive loss using positive and negative pairs, where positive pairs refers to pairs of images which are augmentations of the same image, and negative pairs refer to augmentations sourcing from the rest.

**FIGURE 1:**
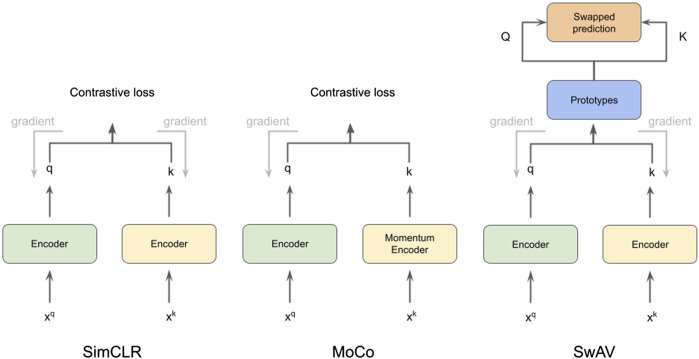
Illustration of methods SimCLR (Chen et al., 2020a), MoCo (He et al., 2020) and SwAV (Zhu et al., 2020), which we use for cryo-ET subtomogram classification.

**FIGURE 2:**
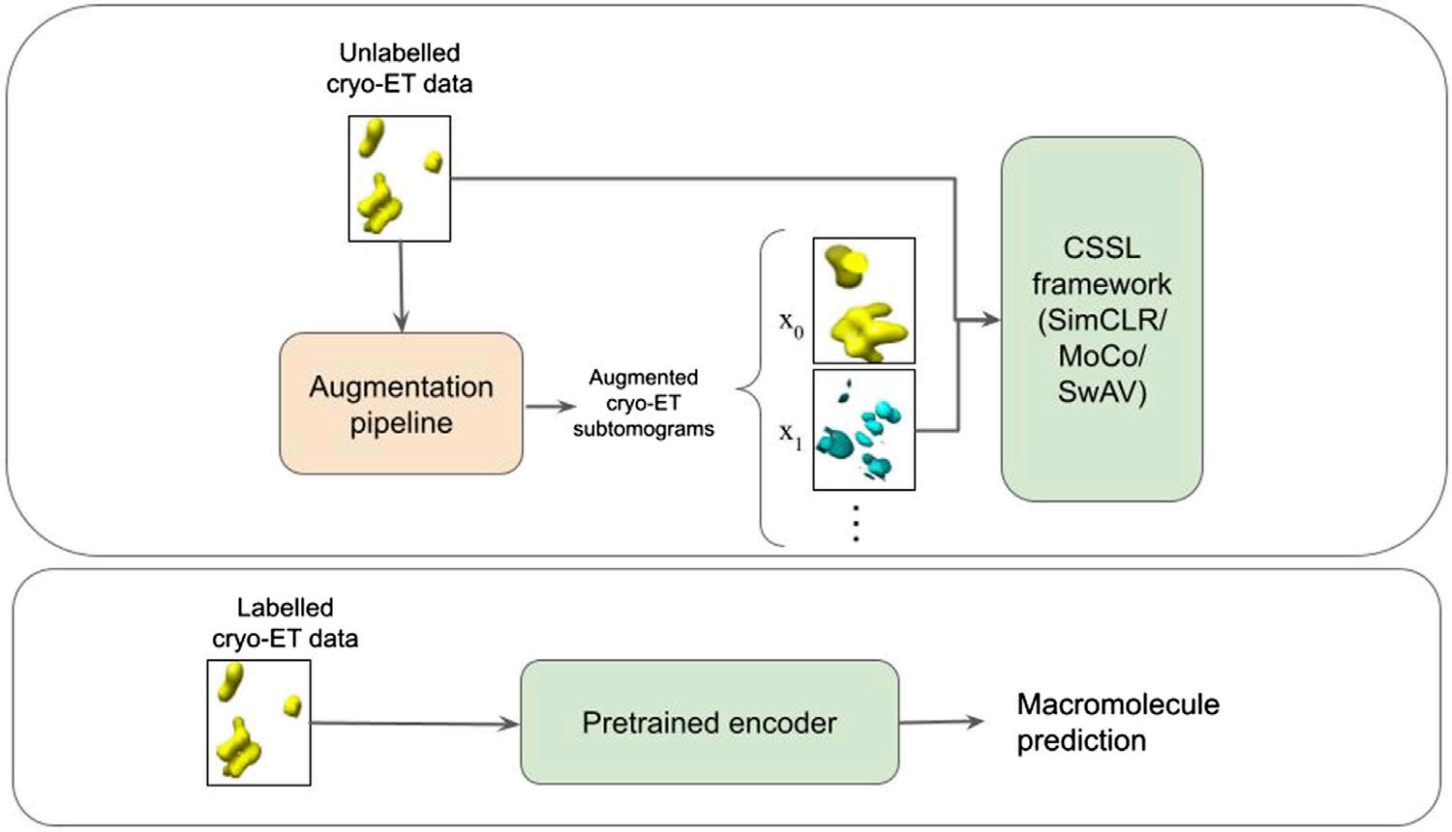
Schematic illustration of the pipeline. The first box represents the CSSL pretraining process. An augmentation pipeline is used to create augmented cryo-ET images which is then fed into a CSSL framework to perform CSSL pretraining. The pretrained encoder is then fine-tuned using labelled cryo-ET data as shown in the second box.

In this work, we use CSSL to improve the current semi-supervised methods for cryo-ET macro-molecule classification. Specifically, we use SimCLR (Chen et al., 2020a), MoCo (He et al., 2020) and SwAV (Zhu et al., 2020) methods to pretrain weights for the classification. These methods are illustrated in Figure 1. To this end, we design a domain-specific augmentation pipeline for cryo-ET data. The augmentation pipeline consists of 3D affine transformations: translation, rotation and scaling. Given the augmentation pipeline, the CSSL task is to contrast positive pairs against negative pairs, enabling a deep-learning model to learn cryo-ET data representations without the need of labels. The CSSL-pretrained weights are then fine-tuned on the downstream classification task using subsets of the training dataset, so as to mimic semi-supervised learning settings. The overall pipeline is shown in Figure 2. The main contributions of this work are summarised as follows:

• We propose a self-supervised learning framework for classification of macromolecules from subtomograms extracted from cryo-ET images.
• We design a simple yet effective data augmentation strategy for 3D cryo-ET subtomogram images.
• We demonstrate the improvements of self-supervised learning in a semi-supervised learning setting using both labelled and unlabelled cryo-ET data.
• Experiments on simulated and experimentally-derived cryo-ET data show the effectiveness and substantial improvements by our proposed approach.

## 2 Related works

### 2.1 Pretraining

The most prominent pretraining approach is supervised pretraining (SP) (Pan and Yang, 2009), where the model solves a supervised task, such as predicting class labels, segmenting images etc., to learn the weight updates. Self-supervised learning (Oord et al., 2018; He et al., 2020; Chen et al., 2020a; Misra and Maaten, 2020), has recently gained promising success as an unsupervised pretraining strategy, even outperforming supervised pretraining in certain applications. Self-supervised pretraining (SSP) solves prediction problems, as is the case with SP. However, unlike SP, the labels which are to be predicted by the model are created from input data, rather than being annotated by human beings.

### 2.2 Data augmentation

Unfortunately, experimental 3D cryo-ET subtomogram image data acquired from cellular imaging, is relatively scarce and hard to collect. Data augmentation is a common method for reducing data bias and helping model generalize better, and can be leveraged to address this issue. Cropping, rotating, occlusion, flipping, shearing, zooming in/out, picture blurring, and adjusting brightness or contrast are all common data-augmentation techniques used in computer vision. In this paper, we propose a brand new data augmentation strategy for 3D cryo-ET subtomogram images, which is especially useful for self-supervised learning.

### 2.3 Self-supervised learning

Self-supervised learning (SSL) has been widely studied to learn better representations of images. SSL generates a loss from a pretext challenge to learn relevant features without the need for human annotations. It only uses the input data to generate auxiliary tasks, allowing deep neural networks to learn effective latent representations by solving them. Numerous methods have been explored for constructing auxiliary tasks, such as temporal correspondence (Wang et al., 2019b; Liu et al., 2019), cross-modal consistency (Wang et al., 2019a), and so on. Rotation prediction (Komodakis and Gidaris, 2018), picture inpainting (Pathak et al., 2016), automated colorization (Zhang et al., 2016), and instance discrimination (Wu et al., 2018) are only a few examples of auxiliary tasks in computer vision.

### 2.4 Semi-supervised learning

Semi-supervised learning techniques utilise both labelled and unlabelled data (Chapelle et al., 2009). Unlabelled data often carry important information which can be leveraged via semi-supervised learning. It is particularly useful in domains where getting labelled data is expensive and time-consuming. Recently, SSL is being increasingly used in conjunction with semi-supervised learning techniques (Zhai et al., 2019; Chen et al., 2020b). SSP is first used to learn data representations from unlabelled data, a process termed as the pretraining phase. The weights learned in the pretraining phase are then fine-tuned for the downstream task using labelled data. Therefore, using SSP, one can utilize both labelled and unlabelled data.

In this paper, to evaluate the effectiveness of SSP in a semi-supervised learning setting, the fine-tuning phase only uses a determined portion of the training set. While in the pretraining phase, which does not require labels, we use the whole training set.

### 2.5 Subtomogram classification

Identifying macromolecules inside cells essentially implies classifying subtomograms extracted from cryo-ET data. Several supervised and semi-supervised methods have been developed for classifying subtomograms. Popular 3D image classification networks (Simonyan and Zisserman, 2014; He et al., 2016; Che et al., 2018) are used for supervised classification. CB3D, DSRF3D_v2 and RB3D (Che et al., 2018) are the recent examples of deep supervised models that have been used. To deal with limited labelled data, active learning (Du et al., 2021) and few shot learning (Yu et al., 2020) based methods have been used to build classification models for classifying macromolecules from subtomograms.

## 3 Methods

The basic flow of our method, demonstrated in Figure 1, is as follows: Perform CSSL, using the specifically designed data-augmentation pipeline with RB3D (Che et al., 2018) as an encoder. For the downstream classification task, we use the CSSL weights to initialize the RB3D architecture and perform supervised classification using the labelled subset of cryo-ET subtomogram images. The steps in our workflow are described in detail in the sequel.

### 3.1 Contrastive self supervised learning techniques

We chose three representative self-supervised learning approaches for our studies: SimCLR (Chen et al., 2020a), MoCo (He et al., 2020), and SwAV (Zhu et al., 2020). All of them are based on contrastive learning (Hadsell et al., 2006). The core principle behind contrastive self-supervised learning is to construct augmented instances from original data samples, design a prediction task that asks if two augmented instances are augmented from a single data sample or not, and train the model by solving this auxiliary task. SimCLR (Chen et al., 2020a) is a simple framework for contrastive learning with bigger batch sizes and considerable data augmentation that yields competitive performance as supervised learning. MoCo (Wu et al., 2018) uses a queue, which holds a dynamic collection of augmented data instances (called keys), to accomplish contrastive learning. For the sake of efficiency, a momentum encoder is used to encode the keys. With a query augmentation, a contrastive loss is defined on the query and keys based on whether they come from the same source. SwAV performs contrastive SSP without requiring computation of pairwise comparisons. In SwAV, clustering is performed on the augmentations of data examples. For cluster assignments for different augmentations from the same image, SwAV encourages them to be consistent. Specifically, the code of one augmentation is predicted based on the representation of another augmentation. Because it does not ask for a big memory bank, this technique is considered to be more efficient interms of memory. We introduce detailed descriptions of contrastive learning for self-supervision and a momentum encoder that is equipped with a queue-structured dictionary in the following sections.

#### 3.1.1 Contrastive learning for self-supervision

Based upon an original subtomogram image from the dataset, CSSL (Hadsell et al., 2006) creates two augmented versions of this image denoted by ***x***_*q*_ and ***x***_*k*_, where ***x***_*q*_ is considered as query and ***x***_*k*_ as key. The query encoder *f*_*q*_ (⋅; *θ*_*q*_) and the key encoder *f*_*k*_ (⋅; *θ*_*k*_), with weights *θ*_*q*_ and *θ*_*k*_ respectively, are adopted to gain latent representations ***q*** = *f*_*q*_ (***x***_*q*_; *θ*_*q*_) and ***k*** = *f*_*k*_ (***x***_*k*_; *θ*_*k*_) for ***x***_*q*_ and ***x***_*k*_.

A positive pair consists of a query and a key from the same image, while a negative pair contains a query and a key from different images. The auxiliary task is designed to tell if the given pair is positive or not.

CSSL employs a queue to hold a collection of keys *k*_*i*_ from different images, and the contrastive loss is computed by:

$$L_{CL}=-\log\frac{\exp\left(q_{j}\cdot k_{j}/\tau \right)}{\exp\left(q_{j}\cdot k_{j}/\tau \right)+\sum_{i}\exp\left(q_{j}\cdot k_{i}/\tau \right)},$$ (1)

with (*q*_*j*_, *k*_*j*_) being a pair obtained from an image instance and *τ* being a temperature parameter (He et al., 2020). During the training process, the encoders are updated by optimizing this loss.

#### 3.1.2 Momentum encoder with queue-structured dictionary

To maintain and perform sampling over key vectors, existing approaches use a variety of strategies (Hadsell et al., 2006; Hjelm et al., 2018; Oord et al., 2018; Chen et al., 2020a). Resorting to the same network *f*_*k*_ = *f*_*q*_ on ***x***_*k*_ and ***x***_*q*_ at the same time, the Siamese-like approach is proposed and has been proven to be effective (Chen X. et al., 2020). However, learning discriminative features from comparing *f*_*k*_ and *f*_*q*_ requires a very big mini-batch size (Chen et al., 2020a). This Siamese-like approach is simple to use, but it is of high computation complexity and is quite resource intensive. As an alternative, a memory bank can be used to store the representations of historical keys in a negative key dictionary *D*_*k*_ = {*k*_*i*_} (Wu et al., 2018). Instead of utilising *f*_*k*_, a mini-batch of keys is sampled from the memory bank at each iteration. The memory bank is updated with the current mini-batch of queries. With an expanded buffer pool, this approach eliminates big batch sizes by default. However, the key sampling step leads to inconsistency when training the encoder. Momentum Contrastive (MoCo) (He et al., 2020) incorporates both types of learning strategies. The memory bank is replaced with a queue-structured key dictionary with a preset length. The oldest key mini-batch will act as the negative key and will be substituted by fresh queries due to the queue’s first-in-first-out (FIFO) principle. This method can avoid negative sampling from being irregular.

An additional important feature of this approach is that parameters of query encoder and key encoder are fixed and do not receive gradient updates. Instead, a running average of the key encoder *f*_*k*_ is used to update the query encoder (Tarvainen and Valpola, 2017; He et al., 2020), referred as *momentum encoder*. Thereby, *θ*_*k*_ and *θ*_*q*_ are updated as follows:

$$\begin{array}{cc} \theta_{q} & \leftarrow \theta_{q}-\alpha \frac{\partial L}{\partial \theta_{q}}\\ \theta_{k} & \leftarrow m\theta_{k}+\left(1-m\right)\theta_{q}, \end{array}$$ (2)

where the momentum coefficient is denoted by *m*, and the query encoder’s learning rate is represented by *α*. As can be seen, *θ*_*q*_ is updated via the back propagating, while *θ*_*k*_ from the key encoder always keeps a running average of previous states.

### 3.2 Encoder

**FIGURE 3:**
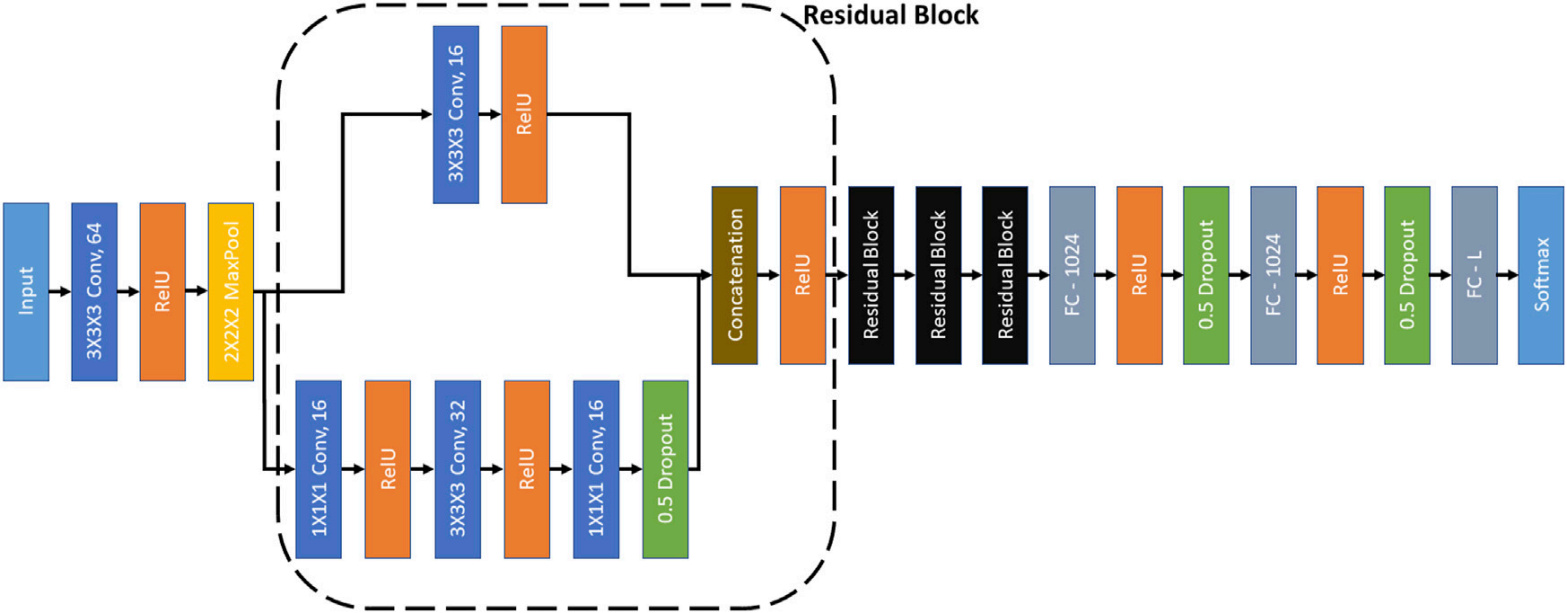
RB3D model (Che et al., 2018). ‘3×3×3 Conv, 64’ represents a 3D convolutional layer with kernel dimensions 3×3×3 and 64 filters. Other convolutional layers follow similar definitions. All the convolutional layers have a stride of 1. ‘2×2×2 MaxPool’ represents a max-pooling operation over the input signal with kernel size 3×3×3 and stride of 2. ‘Concatenation’ denotes the concatenation of the filters of the same dimensions. ‘FC-1024’ represents a fully connected layer with 1024 neurons. The ‘L’ in ‘FC-L’ corresponds to the output dimension. ‘RelU’ and ‘Softmax’ are activation functions.

We have used RB3D (Che et al., 2018) as the encoder in MoCo. The architecture of RB3D is illustrated in Figure 3. RB3D is a 3D residual block based neural network, which was designed specifically for classifying 3D cryo-ET images.

### 3.3 Data-augmentation pipeline

Original data-augmentation pipelines used in CSSL methods such as SimCLR, MoCo and SwAV were designed primarily for traditional 2D RGB image-datasets such as ImageNet (Deng et al., 2009). The augmentation pipeline used random changes in brightness, contrast, saturation and hue of RGB images, along with random horizontal flips and random resized cropping. This augmentation pipeline is very specific to ImageNet like datasets, and needs to be modified to be applied to a different domain (Chaitanya et al., 2020).

Due to the expensive annotation process, experimentally acquired, biological cryo-ET dataset sizes are quite small. Further, the dimensions of subtomograms are also usually small (32^3^ and 28^3^ in the two datasets we use in this paper). In such a case, using strong augmentations can make the pretraining process difficult. We experimented with various permutations and combinations of strong augmentations such as Gaussian blur, Gamma correction, elastic transformations, bias-field etc (Pérez-García et al., 2021). However, due to small-sized datasets coupled with small dimensions of subtomograms, such a augmentation pipeline proved to be too complex for the model to learn useful features during the pretraining phase.

We also considered other image-level augmentations such as the tomography artefacts, e.g. missing wedge effect and electron optical factors [using Contrast Transfer Function (CTF) and Modulation Transfer Function (MTF)], but they are mostly specific to 3D tomography reconstruction from 2D tilt series of cryo-ET images. However, since subtomogram classification is a far downstream task from reconstructing 3D tomograms, it may not be possible to include artefacts that are encountered in a far upstream step in our augmentation pipeline for subtomogram classification. Nevertheless, simulated subtomograms are extracted from the simulated tomograms and, while generating simulated tomograms, we have incorporated the aforementioned tomography artefacts. Consequently, the tomography artefacts are inherent in the simulated subtomogram dataset too. Since the contrastive-learning methods give promising results for simulated data in the presence of tomography artefacts, we consider that contrastive learning methods are robust towards the presence of such artefacts.

Based on the above arguments, we propose a simple yet effective and fine-tuned data-augmentation pipeline as follows:

1) A random resized crop of the image is taken with a probability of 50%. The scale range of the cropped image before resizing is between 0.5 and 1.
2) A random affine transformation is applied with a probability of 50%. This affine transformation includes rotation, translation, and scaling. Image rotation is done by a random angle in the range -45 to 45° along the *z* axis. Horizontal translation of the image is done by a random fraction ≤0.1 of horizontal dimension of the image. Similarly, the vertical translation is done by a random fraction ≤0.1 of the vertical dimension of the image. The image may be scaled up or down by a random scale-factor ≤0.1 .

The intuition behind the above augmentation-pipeline is that to judge if a pair of augmented images originate from a common subtomogram image or not, the model would have to learn global 3D spatial features. These features would then be helpful in downstream classification tasks and may prevent overfitting upon transfer to smaller datasets (Newell and Deng, 2020).

## 4 Experiments

### 4.1 Datasets

#### 4.1.1 Simulated data

**FIGURE 4:**
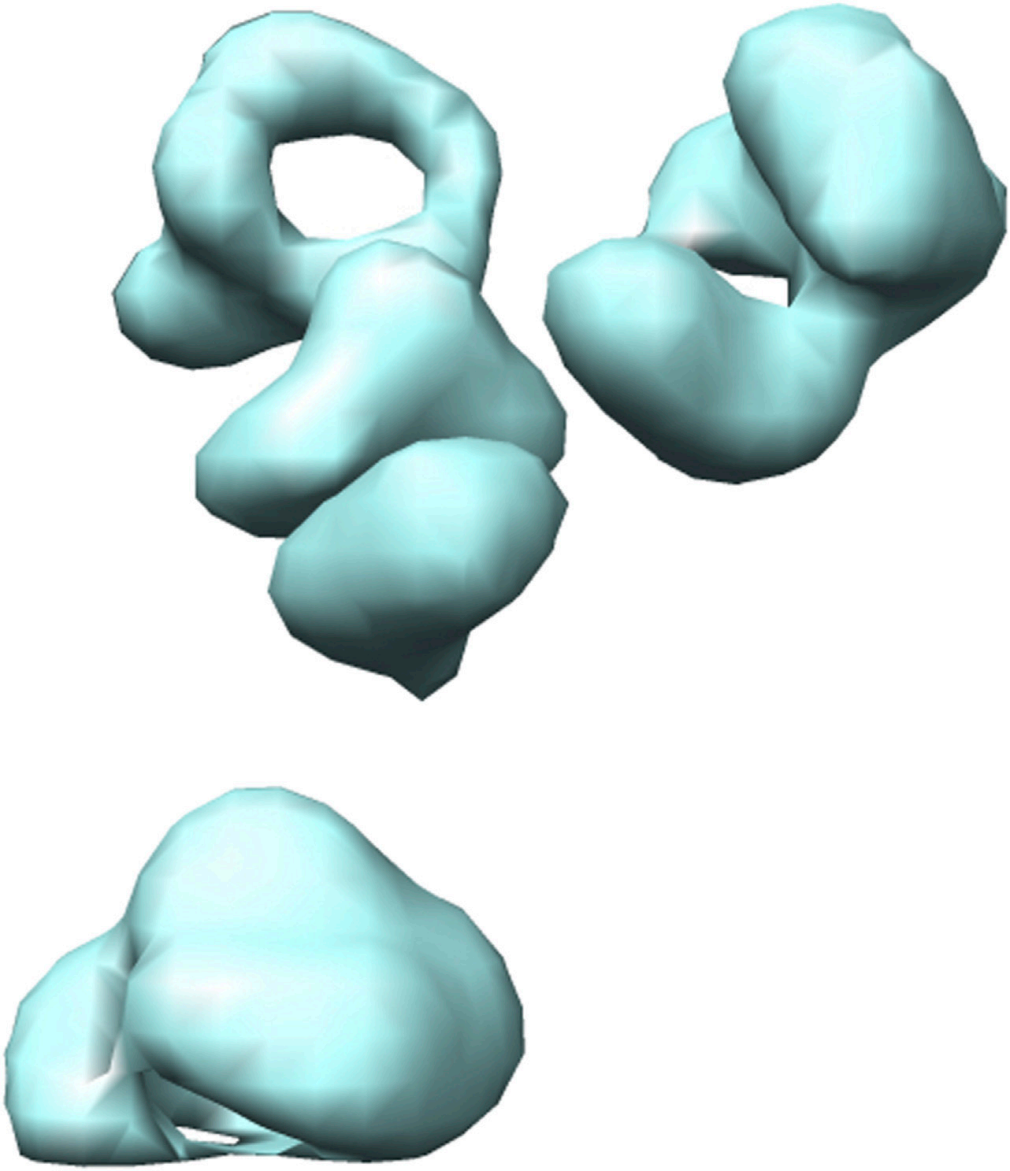
3D isosurface visualization of simulated 2h12 macromolecule, along with randomly simulated macromolecules depicting a crowded subcellular environment.

**FIGURE 5:**
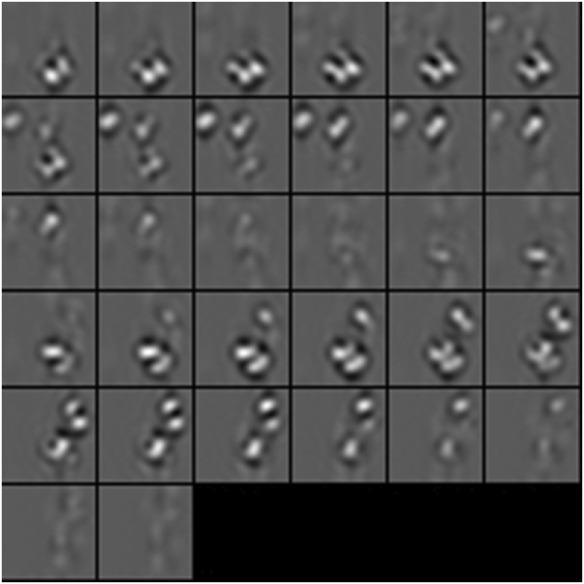
2D subtomogram slice visualization of simulated 2h12 macromolecule, along with randomly simulated macromolecules depicting a crowded subcellular environment.

Several different methods exist for simulating cryo-ET data (Pei et al., 2016; Liu et al., 2020a,b). Here, we use the framework designed by Liu et al. (Liu et al., 2020b). They proposed an efficient gradient descent based method to generate 3D cryo-ET subtomogram images of a target macromolecule with a crowded environment having several random neighbouring macromolecules. The macromolecules are randomly rotated and translated. Further, the simulation procedure includes tomographic artefacts such as the missing wedge effect and electron optical factors to mimic experimentally-acquired cryo-ET images. For illustration, the 3D visualization formed using Chimera (Pettersen et al., 2004) and the 2D slices of a simulated 2h12 macromolecule are shown in Figures 4, 5.

For our experiments, we use three simulated datasets with signal to noise ratio (SNR) as *∞*, 0.05 and 0.03. Each dataset has 500 images per class for 10 classes and each subtomogram is of size 32^3^ (32 × 32 × 32). For our experiments, the three simulated datasets are split in ratio 60:20:20 for training, validation and testing respectively.

#### 4.1.2 Experimentally acquired biological data

**FIGURE 6:**
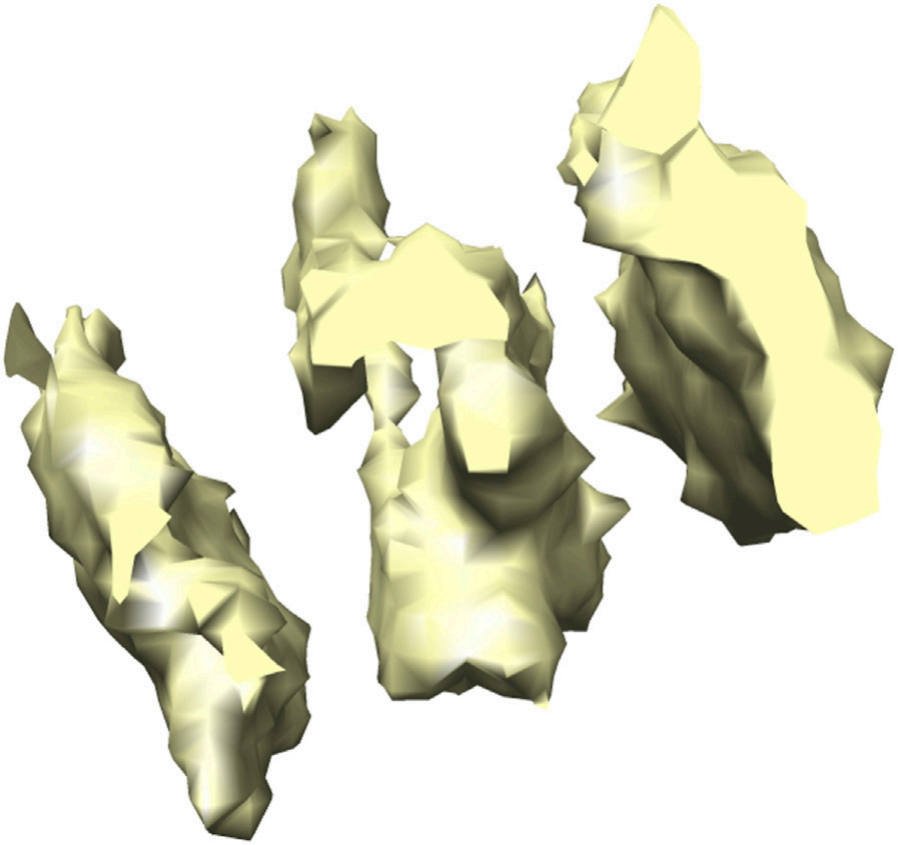
3D isosurface visualization of T20 S proteasome (EMPIAR 10143) macromolecule, extracted from Noble single particle dataset.

**FIGURE 7:**
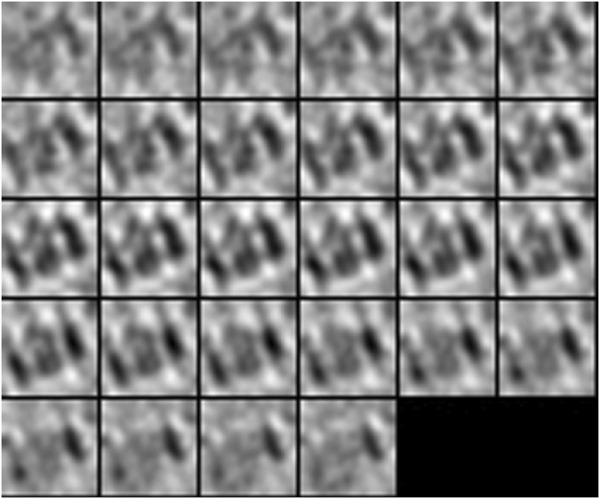
2D subtomogram slice visualization of T20 S proteasome (EMPIAR 10143) macromolecule, extracted from Noble single particle dataset.

The real dataset has been constructed from the Noble single particle dataset (Noble et al., 2018). For each tomogram in the Noble single particle dataset, potential structural regions have been extracted using the Difference-of-Gaussians (DoG) method (Pei et al., 2016). The top 1000 sub-volumes were selected according to cross-correlation scores (Zeng et al., 2018) and then 400 subtomograms were selected manually for each class (Liu et al., 2019). The final constructed dataset has 400 samples for seven classes and each subtomogram is of size 28^3^ (28 × 28 × 28). For illustration, 3D visualization formed using Chimera (Pettersen et al., 2004) and 2D slices of an extracted T20 S proteasome macromolecule is shown in Figures 6, 7. For our experiments, the dataset is split with the ratio of 3:1:1 for training, validation and testing respectively.

### 4.2 Experimental settings

Simulated data: For the MoCo pretraining phase, the MoCo queue size is set to 128. The momentum variable for updating the key encoder is kept as 0.999 and the temperature parameter *τ* is set as 0.2. Adam optimiser (Kingma and Ba, 2014) is used, with learning rate 1*e* ^−4^, weight-decay 1*e* ^−4^ and batch-size 16. The training is done for 200 epochs. For SimCLR and SwAV pretraining phase, the settings are directly inherited from (Chen et al., 2020a) and (Zhu et al., 2020). For the fine-tuning phase, we use the SGD optimiser with cosine decay schedule (Loshchilov and Hutter, 2016). The learning rate is 5*e* ^−4^, weight-decay 1*e* ^−4^ and batch-size 16. The fine-tuning is done for 50 epochs and the model with best validation accuracy is chosen. For normal supervised learning with random initialization, the same hyper-parameters as that of MoCo fine-tuning phase are used.

Experimental biological data: For the MoCo pretraining phase, the hyper-parameters are the same as those for simulated data except for MoCo queue size, which is set as 64. In the fine-tuning phase, for 100 and 75% labelled experiments, the hyper-parameters are same as that for simulated data. For 25 and 50% labelled experiment, the learning rate is 1*e* ^−4^.

### 4.3 Experimental results

For both simulated and real datasets, we randomly select 25, 50, 75 and 100% of the training set size, and then fine-tune the classification models on these subsets. All the experiments are run 5 times and the average accuracy and the standard deviation are reported. Our results for simulated data have been shown in Table 1 and the results for experimental data in Table 2. We found that subtomogram classification accuracy for our experimentally acquired dataset is comparatively higher than the simulated dataset. This is because of the higher complexity of the simulated dataset due to higher resolution and more closely packed macromolecules. As a result of dataset complexity and small training set, the highest accuracy achieved for simulated dataset is around 69%. At the same time, MoCo outperforms the other two CSSL baselines in most experiments. This may be because MoCo extends the idea of contrastive learning by leveraging an extra dictionary along with a momentum encoder, and is more robust and adaptable to be applied to cryo-ET data. We use two-tailed student’s t-test to reject null-hypothesis (Cox, 1982). The *p*-value of the MoCo results obtained is 0.046. Considering significance level, *α* = 0.05, we reject the null hypothesis.

**TABLE 1:** Comparison of subtomogram classification accuracy (%) with standard deviation on experimental biological data. Classifier with CSSL pretrained initial weights performs much better than classifier with random initial weights.

(%)Labelled	SNR	Random init	SimCLR	SwAV	MoCo
100	*∞*	59.1 ± 1.1	64.4 ± 1.0	66.7 ± 2.4	68.6 ± 1.4
	0.05	47.9 ± 2.1	63.1 ± 0.8	65.8 ± 2.1	67.3 ± 0.6
	0.03	47.1 ± 2.1	54.9 ± 1.0	58.8 ± 1.3	57.5 ± 1.7
75	*∞*	37.7 ± 1.1	54.7 ± 1.3	55.4 ± 1.6	59.9 ± 3.1
	0.05	35.7 ± 0.8	54.1 ± 1.5	54.7 ± 1.8	59.6 ± 0.4
	0.03	37.6 ± 0.6	51.8 ± 1.8	52.0 ± 2.3	60.7 ± 0.8
50	*∞*	24.0 ± 0.9	51.4 ± 1.0	50.0 ± 3.0	53.0 ± 1.6
	0.05	23.5 ± 0.7	50.1 ± 0.9	48.9 ± 2.3	49.2 ± 3.1
	0.03	21.7 ± 0.7	49.8 ± 1.7	46.5 ± 3.1	56.5 ± 0.7
25	*∞*	16.0 ± 0.6	37.4 ± 1.0	34.2 ± 1.8	39.3 ± 1.0
	0.05	12.9 ± 1.2	33.9 ± 2.4	34.8 ± 2.9	27.5 ± 1.3
	0.03	15.1 ± 0.8	31.4 ± 1.9	30.5 ± 2.1	30.1 ± 1.7

**TABLE 2:** Comparison of subtomogram classification accuracy (%) with standard deviation on real data. Classifiers with CSSL pretrained initial weights always perform better than classifiers with random initial weights proving the efficacy of CSSL pretraining.

(%)Labelled	Random init	MoCo
100	97.0 ± 0.2	98.5 ± 0.7
75	97.0 ± 0.3	98.6 ± 0.7
50	94.3 ± 1.2	98.2 ± 0.4
25	46.5 ± 0.9	98.4 ± 0.4

**FIGURE 8:**
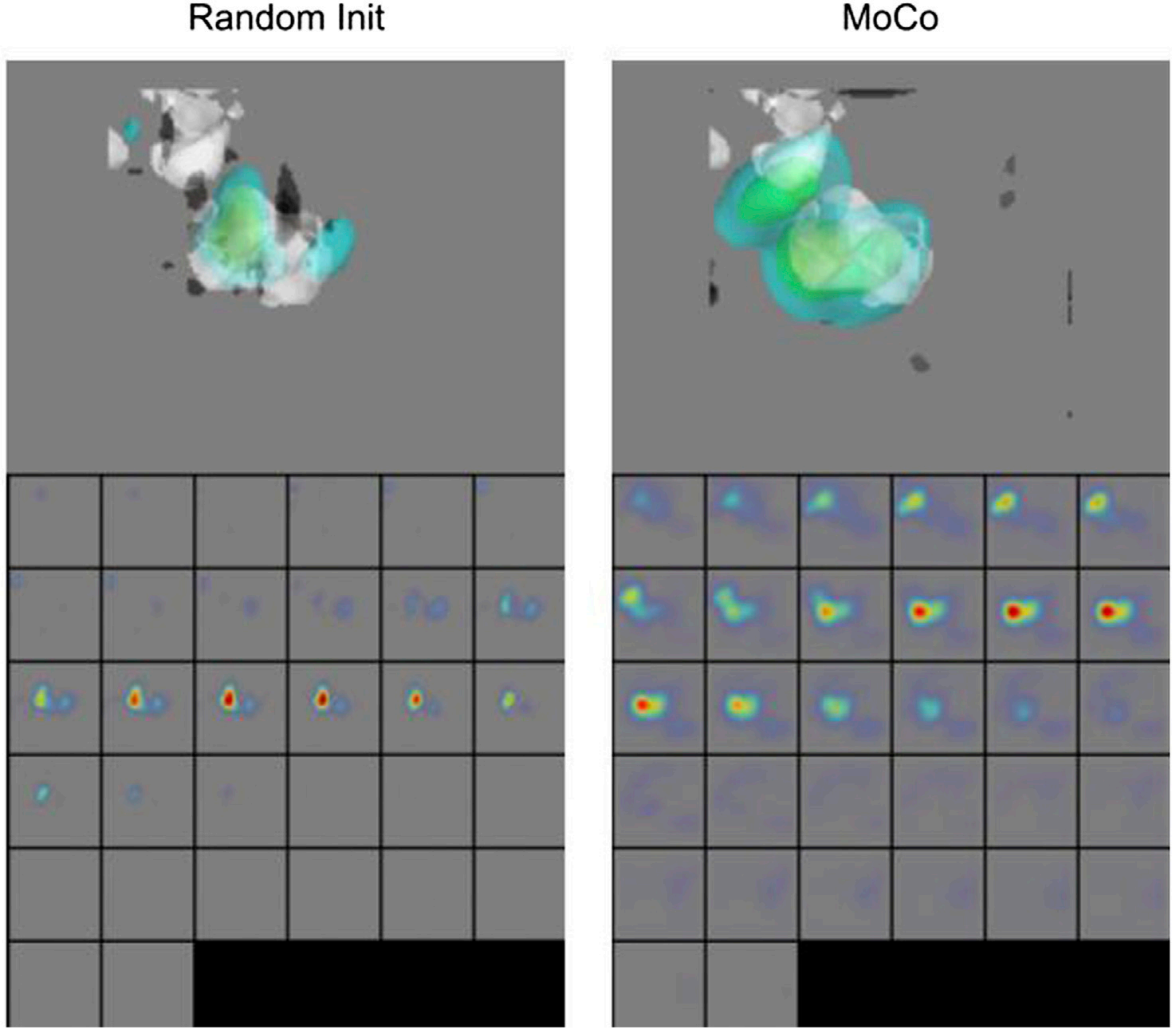
Grad-CAM Visualizations. CSSL (MoCo) pretrained model shows wider regions of 3D space, indicating regularization effect of CSSL pretraining (Newell and Deng, 2020).

We further show the Grad-CAM visualizations (Selvaraju et al., 2017) of a sample subtomogram image for CSSL-pretrained (MoCo) and randomly initialized models in Figure 8, which roughly highlights the region important for making the classification decision. We have used M3d-CAM (Gotkowski et al., 2020) to make these visualizations. It can be observed that the CSSL-pretrained model along with giving higher accuracy also considers wider regions of the 3D environment. These data indicate that CSSL pretraining has a regularization effect on the model (Newell and Deng, 2020). The improvements of CSSL methods over Random Init in all experiments show that the classifier can leverage knowledge gained from CSSL and effectively exploit the representations obtained via pretraining.

## 5 Conclusion

In this paper, we addressed the problem of utilizing unlabelled data for macromolecular structure classification from cryo-ET subtomograms. We developed a pipeline that uses the unlabelled subtomogram data for pretraining weights of a classifier using CSSL methods: SimCLR, MoCo and SwAV, yielding a regularization effect over the classification model. To this end, we designed a unique data-augmentation pipeline for cryo-ET subtomogram data. Our pipeline was able to generate cryo-ET subtomogram images, and those generated images worked well as a source of augmentation for self-supervised learning. We fine-tune the CSSL pretrained weights using labelled subtomograms for the downstream classification task. Taken together, we present a novel workflow that provides significant improvement over traditional classification methods on both simulated and real data.

## Data Availability

The method to generate the datasets used this paper has been described in Section 4.1.

## References

[B1] Best C. Nickell S. Baumeister W. (2007). Localization of protein complexes by pattern recognition. Methods Cell Biol. 79, 615–638. 10.1016/S0091-679X(06)79025-2 17327177

[B2] Caron M. Misra I. Mairal J. Goyal P. Bojanowski P. Joulin A. (2020). Unsupervised learning of visual features by contrasting cluster assignments. Adv. Neural Inf. Process. Syst. 33, 9912–9924.

[B3] Chaitanya K. Erdil E. Karani N. Konukoglu E. (2020). Contrastive learning of global and local features for medical image segmentation with limited annotations. Adv. Neural. Inf. Process Syst. 33, 12546–12558.

[B4] Chapelle O. Scholkopf B. Zien A. (Editors) (2009). Semi-supervised learning. Transactions on Neural Networks. (IEEE Transactions on Neural Networks), 20 (3), 542–542.

[B5] Che C. Lin R. Zeng X. Elmaaroufi K. Galeotti J. Xu M. (2018). Improved deep learning-based macromolecules structure classification from electron cryo-tomograms. Mach. Vis. Appl. 29, 1227–1236. 10.1007/s00138-018-0949-4 31511756 PMC6738941

[B6] Chen T. Kornblith S. Norouzi M. Hinton G. (2020a). “A simple framework for contrastive learning of visual representations,” in International conference on machine learning, PMLR, 1597–1607.

[B7] Chen T. Kornblith S. Swersky K. Norouzi M. Hinton G. (2020b). Big self-supervised models are strong semi-supervised learners Adv. Neural. Inf. Process Syst. 33, 22243–22255.

[B8] Chen X. Fan H. Girshick R. He K. (2020). Improved baselines with momentum contrastive learning. *arXiv preprint arXiv:2003.04297*

[B9] Cox D. R. (1982). Statistical significance tests. Br. J. Clin. Pharmacol. 14, 325–331. 10.1111/j.1365-2125.1982.tb01987.x 6751362 PMC1427620

[B10] Cyrklaff M. Risco C. Fernández J. J. Jiménez M. V. Estéban M. Baumeister W. (2005). Cryo-electron tomography of vaccinia virus. Proc. Natl. Acad. Sci. U. S. A. 102, 2772–2777. 10.1073/pnas.0409825102 15699328 PMC549483

[B11] Deng J. Dong W. Socher R. Li L.-J. Li K. Fei-Fei L. (2009). “Imagenet: A large-scale hierarchical image database,” in Cvpr.

[B12] Du X. Wang H. Zhu Z. Zeng X. Chang Y.-W. Zhang J. (2021). Active learning to classify macromolecular structures *in situ* for less supervision in cryo-electron tomography. Bioinformatics 37, 2340–2346. 10.1093/bioinformatics/btab123 PMC12158443 33620460

[B13] Frazier Z. Xu M. Alber F. (2017). Tomominer and tomominercloud: A software platform for large-scale subtomogram structural analysis. Structure 25, 951–961.e2. 10.1016/j.str.2017.04.016 28552576 PMC5567730

[B14] Gan L. Jensen G. J. (2012). Electron tomography of cells. Q. Rev. Biophys. 45, 27–56. 10.1017/S0033583511000102 22082691 PMC12019784

[B16] Gotkowski K. Gonzalez C. Bucher A. Mukhopadhyay A. (2020). M3d-cam: A pytorch library to generate 3d data attention maps for medical deep learning. *arXiv preprint arXiv:2007.00453*

[B17] Grünewald K. Desai P. Winkler D. C. Heymann J. B. Belnap D. M. Baumeister W. (2003). Three-dimensional structure of herpes simplex virus from cryo-electron tomography. Science 302, 1396–1398. 10.1126/science.1090284 14631040

[B18] Hadsell R. Chopra S. LeCun Y. (2006). “Dimensionality reduction by learning an invariant mapping,” in 2006 IEEE Computer Society Conference on Computer Vision and Pattern Recognition (CVPR’06) (IEEE), 1735–1742.

[B19] Hao Y. Wan X. Yan R. Liu Z. Li J. Zhang S. (2022). Vp-detector: A 3d multi-scale dense convolutional neural network for macromolecule localization and classification in cryo-electron tomograms. Comput. Methods Programs Biomed. 221, 106871. 10.1016/j.cmpb.2022.106871 35584579

[B20] He K. Fan H. Wu Y. Xie S. Girshick R. (2020). “Momentum contrast for unsupervised visual representation learning,” in Proceedings of the IEEE/CVF conference on computer vision and pattern recognition, 9729–9738.

[B21] He K. Zhang X. Ren S. Sun J. (2016). “Deep residual learning for image recognition,” in Proceedings of the IEEE conference on computer vision and pattern recognition, 770–778.

[B22] Hjelm R. D. Fedorov A. Lavoie-Marchildon S. Grewal K. Bachman P. Trischler A. (2018). Learning deep representations by mutual information estimation and maximization. *arXiv preprint arXiv:1808.06670*

[B23] Kingma D. Ba J. (2014). “Adam: A method for stochastic optimization,” in International Conference on Learning Representations. PMC13460018 42583277

[B24] Klein S. Cortese M. Winter S. L. Wachsmuth-Melm M. Neufeldt C. J. Cerikan B. (2020). Sars-cov-2 structure and replication characterized by *in situ* cryo-electron tomography. Nat. Commun. 11, 5885–5910. 10.1038/s41467-020-19619-7 33208793 PMC7676268

[B15] Komodakis N. Gidaris S. (2018). “Unsupervised representation learning by predicting image rotations,” in International Conference on Learning Representations (ICLR).

[B25] Koning R. I. Koster A. J. (2009). Cryo-electron tomography in biology and medicine. Ann. Anatomy-Anatomischer Anzeiger 191, 427–445. 10.1016/j.aanat.2009.04.003 19559584

[B27] Liu S. Ban X. Zeng X. Zhao F. Gao Y. Wu W. (2020a). A unified framework for packing deformable and non-deformable subcellular structures in crowded cryo-electron tomogram simulation. BMC Bioinforma. 21, 1–24. 10.1186/s12859-020-03660-w PMC7488303 32907544

[B28] Liu S. Ma Y. Ban X. Zeng X. Nallapareddy V. Chaudhari A. (2020b). “Efficient cryo-electron tomogram simulation of macromolecular crowding with application to sars-cov-2,” in 2020 IEEE International Conference on Bioinformatics and Biomedicine (BIBM), 80. 10.1109/BIBM49941.2020.9313185

[B26] Liu S. Du X. Xi R. Xu F. Zeng X. Zhou B. (2019). Semi-supervised macromolecule structural classification in cellular electron cryo-tomograms using 3d autoencoding classifier. BMVC 30. 10.5244/C.33.67

[B30] Loshchilov I. Hutter F. (2016). Sgdr: Stochastic gradient descent with warm restarts. *arXiv preprint arXiv:1608.03983*

[B31] Lučić V. Rigort A. Baumeister W. (2013). Cryo-electron tomography: The challenge of doing structural biology *in situ* . J. Cell Biol. 202, 407–419. 10.1083/jcb.201304193 23918936 PMC3734081

[B32] Misra I. Maaten L. v. d. (2020). “Self-supervised learning of pretext-invariant representations,” in Proceedings of the IEEE/CVF Conference on Computer Vision and Pattern Recognition, 6707–6717.

[B33] Moebel E. Kervrann C. (2022). Towards unsupervised classification of macromolecular complexes in cryo electron tomography: Challenges and opportunities. Comput. Methods Programs Biomed. 225, 107017. 10.1016/j.cmpb.2022.107017 35901628

[B34] Murata K. Wolf M. (2018). Cryo-electron microscopy for structural analysis of dynamic biological macromolecules. Biochim. Biophys. Acta. Gen. Subj. 1862, 324–334. 10.1016/j.bbagen.2017.07.020 28756276

[B35] Newell A. Deng J. (2020). “How useful is self-supervised pretraining for visual tasks?,” in Proceedings of the IEEE/CVF Conference on Computer Vision and Pattern Recognition, 7345–7354.

[B36] Noble A. J. Dandey V. P. Wei H. Brasch J. Chase J. Acharya P. (2018). Routine single particle cryoem sample and grid characterization by tomography. Elife 7, e34257. 10.7554/eLife.34257 29809143 PMC5999397

[B37] Noroozi M. Favaro P. (2016). “Unsupervised learning of visual representations by solving jigsaw puzzles,” in European Conference on Computer Vision (Springer), 69–84.

[B38] Oikonomou C. M. Jensen G. J. (2017). Cellular electron cryotomography: Toward structural biology *in situ* . Annu. Rev. Biochem. 86, 873–896. 10.1146/annurev-biochem-061516-044741 28426242

[B39] Oord A. v. d. Li Y. Vinyals O. (2018). Representation learning with contrastive predictive coding. *arXiv preprint arXiv:1807.03748*

[B40] Pan S. J. Yang Q. (2009). A survey on transfer learning. IEEE Trans. Knowl. Data Eng. 22, 1345–1359. 10.1109/tkde.2009.191

[B41] Pathak D. Krahenbuhl P. Donahue J. Darrell T. Efros A. A. (2016). “Context encoders: Feature learning by inpainting,” in Proceedings of the IEEE conference on computer vision and pattern recognition, 2536–2544.

[B42] Pei L. Xu M. Frazier Z. Alber F. (2016). Simulating cryo electron tomograms of crowded cell cytoplasm for assessment of automated particle picking. BMC Bioinforma. 17, 405–413. 10.1186/s12859-016-1283-3 PMC5050594 27716029

[B43] Pérez-García F. Sparks R. Ourselin S. (2021). Torchio: A python library for efficient loading, preprocessing, augmentation and patch-based sampling of medical images in deep learning. Comput. Methods Programs Biomed. 208, 106236doi. 10.1016/j.cmpb.2021.106236 34311413 PMC8542803

[B44] Pettersen E. F. Goddard T. D. Huang C. C. Couch G. S. Greenblatt D. M. Meng E. C. (2004). Ucsf chimera—A visualization system for exploratory research and analysis. J. Comput. Chem. 25, 1605–1612. 10.1002/jcc.20084 15264254

[B45] Selvaraju R. R. Cogswell M. Das A. Vedantam R. Parikh D. Batra D. (2017). “Grad-cam: Visual explanations from deep networks via gradient-based localization,” in Proceedings of the IEEE international conference on computer vision, 618–626.

[B46] Simonyan K. Zisserman A. (2014). Very deep convolutional networks for large-scale image recognition. *arXiv preprint arXiv:1409.1556*

[B47] Tarvainen A. Valpola H. (2017). “Mean teachers are better role models: Weight-averaged consistency targets improve semi-supervised deep learning results,” in NIPS.

[B48] Tian Y. Krishnan D. Isola P. (2019). “Contrastive multiview coding,” in European conference on computer vision. (Cham: Springer), 776–794.

[B49] Wang X. Huang Q. Celikyilmaz A. Gao J. Shen D. Wang Y.-F. (2019a). “Reinforced cross-modal matching and self-supervised imitation learning for vision-language navigation,” in Proceedings of the IEEE Conference on Computer Vision and Pattern Recognition, 6629–6638.

[B50] Wang X. Jabri A. Efros A. A. (2019b). “Learning correspondence from the cycle-consistency of time,” in Proceedings of the IEEE Conference on Computer Vision and Pattern Recognition, 2566–2576.

[B51] Wu Z. Xiong Y. Yu S. X. Lin D. (2018). “Unsupervised feature learning via non-parametric instance discrimination,” in Proceedings of the IEEE Conference on Computer Vision and Pattern Recognition, 3733–3742.

[B52] Yu L. Li R. Zeng X. Wang H. Jin J. Yang G. (2020). Few shot domain adaptation for *in situ* macromolecule structural classification in cryo-electron tomograms. Bioinformatics 37, 185–191. 10.1093/bioinformatics/btaa671 PMC8237843 32722755

[B53] Zeng X. Leung M. R. Zeev-Ben-Mordehai T. Xu M. (2018). A convolutional autoencoder approach for mining features in cellular electron cryo-tomograms and weakly supervised coarse segmentation. J. Struct. Biol. 202, 150–160. 10.1016/j.jsb.2017.12.015 29289599 PMC6661905

[B54] Zhai X. Oliver A. Kolesnikov A. Beyer L. (2019). “S4l: Self-supervised semi-supervised learning,” in Proceedings of the IEEE/CVF International Conference on Computer Vision, 1476–1485.

[B55] Zhang P. (2013). Correlative cryo-electron tomography and optical microscopy of cells. Curr. Opin. Struct. Biol. 23, 763–770. 10.1016/j.sbi.2013.07.017 23962486 PMC3812453

[B56] Zhang R. Isola P. Efros A. A. (2016). “Colorful image colorization,” in European conference on computer vision (Springer), 649–666.

[B57] Zhu Z. Wang Y. Zhou X. Yang L. Meng G. Zhang Z. (2020). Swav: A web-based visualization browser for sliding window analysis. Sci. Rep. 10, 149–154. 10.1038/s41598-019-57038-x 31924845 PMC6954255

